# Genito-Urinary Surgery

**Published:** 1894-08-18

**Authors:** 


### PROGRESS IN SURGERY.

### GENITO-URINARY SURGERY.

**Surgery of Prostate.**—In the *Lancet* of April 14th Dr. Nicoll[1] reviews the question of prostatectomy, points out that its chief dangers are from hæmorrhage and septic diseases, that insufficient operations may mar the result or permanent injury be done by lacerations of parts adjacent to the prostate. To avoid these evils he recommends the following procedure: (1) Suprapubic cystotomy followed by careful cleansing of the bladder; (2) the prostate is reached from a perineal incision and enucleated. Details of the operation and after treatment are given. Legueu[2], in recording a case of removal of a prostatic growth, points out the risk of loss of power of contraction of the bladder from a thickening and inelastic condition of the vesical walls which necessitated the use of a catheter even after the operation. Bier[3] has ligatured the internal iliac arteries to induce atrophy of the prostate and reports favourably upon the method. Griffiths[4] gives particulars of Ramm's cases of bilateral castration for prostatic enlargement in which almost immediate atrophy of the prostate with relief to the bladder trouble followed the operation. Chance[5] advocates removal of portions of the prostate through a suprapubic opening by means of a flushing gouge, and urges the propriety of early operation. In the discussion following the reading of Mr. Chance's paper, Mr. Kendal Franks, as well as Mr. Chance, advocates supplementary perineal drainage. Moullin,[6] in discussing operations on the prostate, advises that prostatectomy should not be performed shortly after an attack of acute retention, on the ground that it is impossible at such time to say how much of the enlargement may subside on the adoption of measures calculated to relieve congestion, and also because at such time the hæmorrhage may be severe. He also suggests the performance of the operation in two stages in cases of septic cystitis, the bladder being opened and cleansed a few days before the gland is removed. He urges free removal if any is taken away at all, on the ground that shrinking following partial removal is an uncertain matter.

**Diseases of the Testicle.**—Balzer and Lacour[7] have successfully treated orchitis by the application of guiacol ointment, one to six of vaseline. Chauffard and Marie[8] speak well of salicylate of soda in doses of ʒiss. to ʒii. in the day. Lauenstein,[9] writing on torsion of the testicle, states that the occurrence of torsion is favoured by (1) a flattened form of the testicle; (2) its "horizontal inversion."

**Diseases of the Bladder.**—Bardenheuer[10] records his third case of excision of the mucous lining of the bladder for tuberculous disease. The difficulties appear to lie in the question of diagnosis, and in that of the restoration of the bladder lining. It may be pointed out that very considerable power of retention is preserved even after nearly complete sloughing away of the mucous lining.

Barling[11] discusses the operative treatment of stone in the bladder in young males. Wheelton Hind[12] records a case of suppression of urine from impacted calculus in a single kidney, the other having been destroyed by disease. The case was not successful as regards saving life, but the obstruction was relieved and urine flowed freely. Purcell[13] relates a case in which part of the bladder was found in an inguinal hernia and was incised and subsequently removed, with a good result. Harry Fenwick[14] mentions a case in which he three times removed an epithelimatous growth from the bladder, once by perineal incision and twice by the suprapubic route; the patient recovered. Symonds[15] records a case in which an epitheloma of the bladder extended forward along the whole length of the urethra. Harry Fenwick[16] suggests that in certain cases of enlarged prostate the difficulties and dangers of sounding by the urethra are so great that it would be wise to pass a trocar into the bladder and pass a sound through the canula, and thus explore. In a similar fashion, by an electric cystoscope the interior of the bladder might be examined. Reginald Harrison[17] discusses the use of perineal lithotrity with special lithotrites in certain cases. Rollet[18] reports a case of his own, and refers to those of other surgeons to show that occasionally the peritoneum in front of the bladder reaches down, and adheres to the pubes, and so is more than ordinarily liable to be wounded in operations.

**Surgery of the Ureter.**—Cotterell[19] relates the case of a woman of 61 from whose ureter he removed a calculus impacted at a point just below the level of the brim of the pelvis. The ureter was exposed by an incision like that used for ligature of the common iliac artery; the wound in the ureter was not closed. In a second case two calculi were removed by an incision in the wall of the vagina after their position had been made out by examination per urethram. Godlee[20] mentions other cases of calculi impacted in the ureter. Henry Morris[21] advocates removal of the kidney, leaving the calculus in the ureter if the kidney is damaged. Page[22] in a valuable paper on rupture of the ureter discusses the symptoms in his own and other recorded cases, pointing out that it may be a considerable time, at least many days, before any characteristic signs of the injury appear. The amount of blood in the urine is usually very small, and may readily escape notice. Abdominal tenderness followed by localised swelling with diminution of the quantity of urine passed after an injury such as would cause rupture of the ureter are the main symptoms. If the swelling is aspirated the fluid drawn off will probably consist of very dilute urine. No treatment appears to be adequate except nephrectomy, which should certainly be performed as soon as any septic disturbance becomes evident. The possibility of suture of the ureter is discussed, and cases are given, while the difficulties of such a proceeding are pointed out, and the close adhession of the ureter to the peritoneum, so that it is turned forwards in stripping the peritoneum from the posterior abdominal wall is commented upon.

**Surgery of the Urethra.**—Bryant[23] reports a case of prolapse of the female urethra in a child. Ward Cousins[24] describes and figures a dilator for the female urethra. The instrument is constructed on the principle of a conical indiarubber distensible bag fitted over a perforated metal stem. The inventor has used it with success. Reclus[25] records a case where the injection of 5 drachms of 5 per cent. solution of cocaine into the urethra was immediately fatal to an old man. Anderson[26] adds another case to the list of successful primary sutures of the ruptured urethra. [It is an operation that should undoubtedly always be at least attempted if catheterism fails, and in the future will very probably become the rule in all cases of ruptured urethra. (Abstractor.)] McGrath[27] advocates the use of large injections of weak solutions of nitrate of silver in chronic urethritis. He injects 12 to 14 oz. of a solution of ¼ gr. to the oz., and then allows the patient immediately to void it again, thus washing out the urethra from behind forwards. Reich[28] records a case of acute inflammation of a seminal vesicle ("spermatocystitis") after gonorrhœa, treated by incision. The vesicle was reached by a perineal incision, the coccyx being resected and the rectum pushed over to the left.

**Syphilis.**—H. Mandel[29] considers the question of syphilitic phlebitis, and points out that it may occur in either a secondary or tertiary lesion. The former occurs in the veins of the extremities, the latter, according to the author, has only been twice described. Both cases were recorded by Von Langenbach, one a gumma of the jugular, the other of the femoral vein.

[1] Lancet, April 14th, 1894. [2] Edin. Med. Jour., May, 1894. [3] Wiene Klin. Woch, 1893, No. 32; Edin. Med. Jour., May, 1894. [4] Brit. Med Jour., May 12th, 1894. [5] Dublin Quarterly Jour. of Med. Sc., April, 1894. [6] Lancet, April 21st, 1894. [7] Lancet, April 21st, 1894. [8] Med. Week, April 13th, 1894. [9] Glas. Med. Jour., May, 1894. [10] Brit. Med. Jour. Epitome, May 5th, 1894. [11] Brit. Med. Jour., May 5th, 1894. [12] Brit. Med. Jour., May 5th, 1894. [13] Lancet, May 5th, 1894. [14] Med. Week, April 6th, 1894. [15] Lancet, April 21st, 1894. [16] Brit. Med. Jour., April 21st, 1894. [17] Lancet, April 7th, 1894. [18] Therap. Gaz., Mar. 15th, 1894. [19] Lancet, May 12th, 1894. [20] Lancet, May 12th, 1894. [21] Lancet, May 12th, 1894. [22] Ann. of Surgery, May, 1894. [23] Med. Week, May 11th, 1894. [24] Brit. Med. Jour., April 7th, 1894. [25] Med, Week, Mar. 30th, 1894. [26] Lancet, June 2nd, 1894. [27] Med. Rec., April 14th, 1894. [28] Med. Week, May 25th, 1894. [29] Med. Week, May 25th, 1894.

